# Out-of-Hospital Cardiac Arrest With Bilateral Urinary Tract Injury Resulting From Cardiopulmonary Resuscitation: A Case Report

**DOI:** 10.7759/cureus.66403

**Published:** 2024-08-07

**Authors:** Shuhei Tada, Shota Kikuta, Shigenari Matsuyama, Satoshi Ishihara

**Affiliations:** 1 Anesthesia and Critical Care, Tokyo Bay Urayasu Ichikawa Medical Center, Urayasu, JPN; 2 Emergency and Critical Care Medicine, Hyogo Emergency Medical Center, Kobe, JPN

**Keywords:** complications (cpr), gross hematuria, chest compression, cardiopulmonary resuscitation, urinary tract injury

## Abstract

A man in his 70s suffered cardiac arrest, and his family initiated cardiopulmonary resuscitation after placing an emergency call. The initial waveform of the automated external defibrillator performed by emergency medical technicians revealed ventricular fibrillation. The patient received cardiovascular life support, including direct current countershock, and was transported to the hospital. Upon arrival, he underwent extracorporeal cardiopulmonary resuscitation using an automated chest compression device. Additionally, an intra-aortic balloon pumping was introduced after coronary angiography and percutaneous coronary intervention. Plain computed tomography images revealed leakage of the contrast medium used during coronary angiography in the bilateral renal pelvis and perirenal area as well as bladder retention. Furthermore, a urine test revealed gross hematuria. There were no findings of prostatic hypertrophy or urinary tract disease. Based on the patient's clinical course, injury caused by chest compression was the most likely etiology of urinary tract injury, which must be considered in such patients. The patient was discharged with cerebral performance category 1, without any complication except urinary tract.

## Introduction

Cardiopulmonary resuscitation (CPR) is an important topic in emergency medicine recently [1]. Chest compressions of proper depth, approximately 5 cm, are considered necessary to achieve good-quality chest compressions [2,3], as the guidelines recommend that chest compressions be performed without hesitation when cardiac arrest is suspected [4,5]. Thus, high-quality chest compressions are critical during CPR; however, they may lead to medical complications [6,7]. The common complications include rib fractures, pneumothorax, thoracic deformity, sternal fracture, and cardiac rupture; however, rare non-chest complications such as gastric rupture, liver injury, and abdominal emphysema have also been reported. We herein describe a case of out-of-hospital cardiac arrest with bilateral urinary tract injury resulting from CPR.

## Case presentation

A 72-year-old man with no specific medical or family history complained of chest pain and lost consciousness approximately 40 minutes before arriving at our hospital. His family witnessed his collapse and called a dispatch center of the fire department for emergency medical services (EMS). His family started chest compressions under telephone guidance by the dispatch center. When the EMS personnel arrived, they found rhythm as ventricular fibrillation (VF) after applying an automated external defibrillator; thus, they performed CPR and defibrillation. The patient achieved a return of spontaneous circulation (ROSC) 16 minutes after loss of consciousness, though he remained apneic. To secure the airway immediately and to prevent aerosol diffusion, a laryngeal tube was inserted, and transport commenced shortly. VF recurred during transport, requiring a second defibrillation; ROSC was successfully achieved thereafter; the second low-flow time was 12 minutes. However, the patient fell into cardiac arrest (VF) again two minutes before arrival at the hospital.

**Figure 1:**
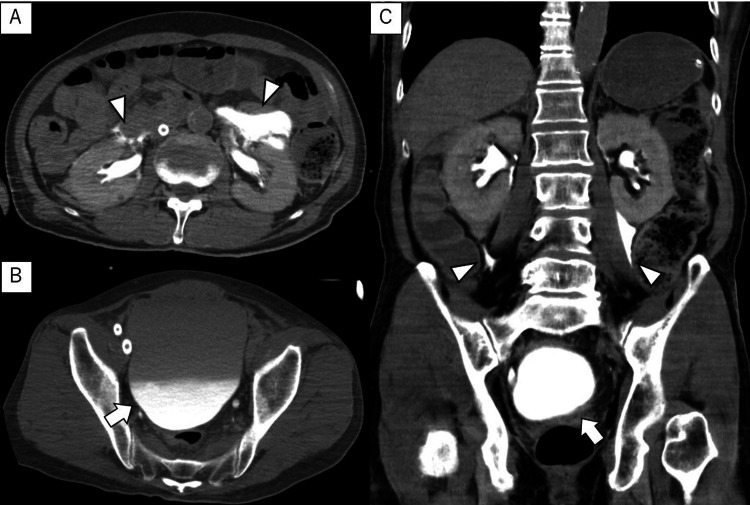
CT imaging before transferring to the ICU Axial and coronal contrast-enhanced CT imaging before the patient was transferred to the ICU showed the leakage of the contrast medium used during CAG in the bilateral renal pelvis and perirenal region (white arrowhead), along with urinary retention (white arrow) CT: computed tomography; ICU: intensive care unit; CAG: coronary angiography

Initial medical treatment was administered in the angiography room. An automatic chest compression device was attached to the patient, and extracorporeal CPR (ECPR) was initiated. Furthermore, veno-arterial extracorporeal membrane oxygenation (VA-ECMO) was introduced by cardiologists and emergency physicians from the right femoral artery and vein 14 minutes after arrival. ROSC was achieved simultaneously with the establishment of ECMO, indicating the duration of arrest was 16 minutes since the third collapse. After intubation, coronary angiography (CAG) and percutaneous coronary intervention were performed due to stenosis in the left circumflex artery and right coronary artery. Under fluoroscopy, a central venous catheter was inserted from the right internal jugular vein, and an intra-aortic balloon pumping (IABP) was placed from the left brachial artery as the left femoral artery was completely occluded due to arteriosclerosis. Before admission to the intensive care unit (ICU), urine occult blood test was strongly positive, and gross hematuria was observed. Plain computed tomography (CT) images were obtained to rule out CPR-related or ECPR-related complications. CT images showed leakage of the contrast medium used during CAG in the bilateral renal pelvis and perirenal region, along with urinary retention (Figure 1A, 1B, 1C). There were no obvious signs of prostatic hypertrophy or malignancy of the urinary tract system, and no rib fractures or sternal fractures were observed. The patient's skin surface had no sign of trauma. Laboratory tests showed that creatinine and eGFR were within normal limits.

The gross hematuria was observed; however, CT revealed good contrast to the distal portion of the ureter. After consulting with the urologist, the patient was conservatively treated for incomplete rupture of the renal pelvis and ureter.

The patient was admitted to the ICU for temperature control and neurointensive care.

**Figure 2:**
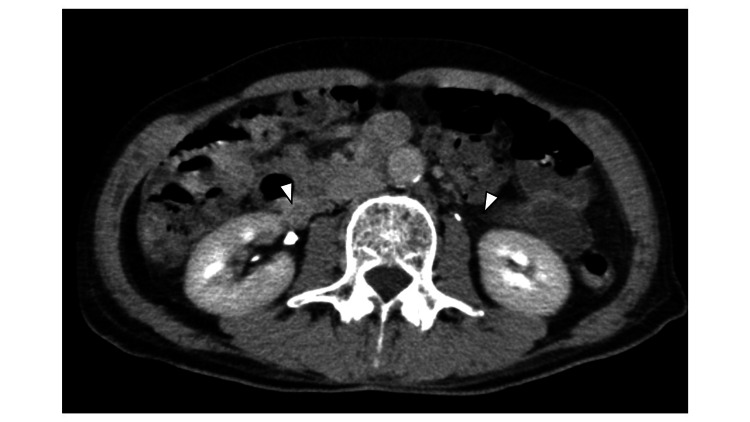
Follow-up axial contrast-enhanced abdominal CT imaging showing no ureteral overflow A follow-up contrast-enhanced CT was performed on day 9 and revealed no ureteral overflow (white arrowhead) CT: computed tomography

On day 1 in the ICU, the temperature was kept at 36.5℃. On day 3, the patient was successfully weaned from IABP and VA-ECMO and both were removed. On day 6, the patient regained consciousness and was extubated. Then on day 9, a follow-up contrast-enhanced CT was performed and revealed no ureteral overflow (Figure 2).

The patient was discharged on day 31 with cerebral performance category 1, without any complication except urinary tract. According to his statement after regaining consciousness, there had been no trauma episode or hematuria before the cardiac arrest.

## Discussion

We experienced an extremely rare case of out-of-hospital cardiac arrest with bilateral urinary tract injury resulting from CPR. Although conservative treatment was chosen and the survey did not lead to a thorough urological examination, the clinical course implied a urinary tract injury due to chest compression. To the best of our knowledge, this is the first report to document that chest compression provoked urinary system injury, let alone bilateral ureteral injury.

Buschmann and Tsokos reported on post-resuscitation organ injuries as complications of CPR. In their review, liver, spleen, and stomach lesions were described as common abdominal injuries, whereas urinary tract injuries were not mentioned [8]. In another prospective multicenter trial, including 222 patients (83 manual CPR and 139 mechanical CPR), no urinary tract injury was reported [9]. Contrary to the liver, stomach, and duodenum, which are located in the middle of the peritoneal cavity and are posteriorly supported by the spine, the kidneys are located at both lateral sides in the retroperitoneal cavity where compressing forces are less transmitted [10]. That must be a reason why renal injuries associated with CPR, particularly bilateral urinary tract injuries, are very rare. This presumption is supported by the case reporting renal injury by CPR in a horseshoe kidney [10]. Moreover, as the renal pelvis is closer to the midline of the trunk than the renal parenchyma, it is consistent with the presumption that CPR damaged the renal pelvis rather than the parenchyma.

There have been several case reports of spontaneous renal pelvis rupture [11,12]. In those reports, urinary retention due to some obstructive mechanism such as malignancy or urinary calculi was remarked [11]. Furthermore, a case of bilateral peripelvic extravasation of urine during enhanced CT has been reported. It suggests that a rapid rise of intraureteral pressure by contrast agent caused seeping out of the renal pelvis. In our case, whether the patient had hydronephrosis before cardiac arrest was not known. However, CT following VA-ECMO revealed prominent intravesical urinary retention. Consequently, we suspect that the retention partly contributed to the urinary tract injury.

Organ damage by chest compressions can result from direct and/or indirect mechanisms. Direct mechanism occurs by the incorrect position of compression, mainly on the epigastric region. In a prospective randomized clinical trial using mechanical chest compression, serious or life-threatening visceral rupture was more observed in 20 of 211 patients (9.5%), compared to eight of 126 manual controls (6.4%) [13]. Moreover, it has been reported that complications may occur due to abnormal positioning [14]. Unlike manual chest compressions, once mechanical chest compression is initiated at an inappropriate position, it can be hardly noticed and rarely corrected. In this case, an automatic chest compression device was used after arrival at our hospital, while the patient's family and EMS personnel performed manual chest compressions. Although it cannot be excluded that the positioning of chest compressions by his family was wrong, it is more possible that the placement of the automatic chest compression device was mistaken and that caused the urinary tract injury. The indirect mechanism denotes the secondary elevation of intra-abdominal pressure caused by intrathoracic pressure elevation due to chest compression. Either direct or indirect mechanism could raise intraureteral pressure by chest compressions.

In the present case, the CT scan failed to show the cause of hematuria but revealed cannulation of ECMO could be performed uneventfully. Based on these findings, the urinary tract injury was quite unlikely precipitated by trauma or iatrogenic complication. In addition, we have speculated that renal tract injury was not serious, because leakage of contrast agent was not shown by CT nine days after without any surgical intervention.

## Conclusions

Spontaneous ureteral injury can be complicated with chest compression. In case of hematuria during the management of patients with cardiac arrest, it might be useful that physicians suspect urinary tract injury.
